# Supplementary material for: Differentiating Human Pluripotent Stem Cells to Cardiomyocytes Using Purified Extracellular Matrix Proteins
Source: Bioengineering (Basel). 2022 Nov 22;9(12):720. doi: 10.3390/bioengineering9120720 (PMC9774171; doi:10.3390/bioengineering9120720)
Supplement: Supplementary file 1 [file bioengineering-09-00720-s001.zip › bioengineering-2022376-supplementary.pdf]

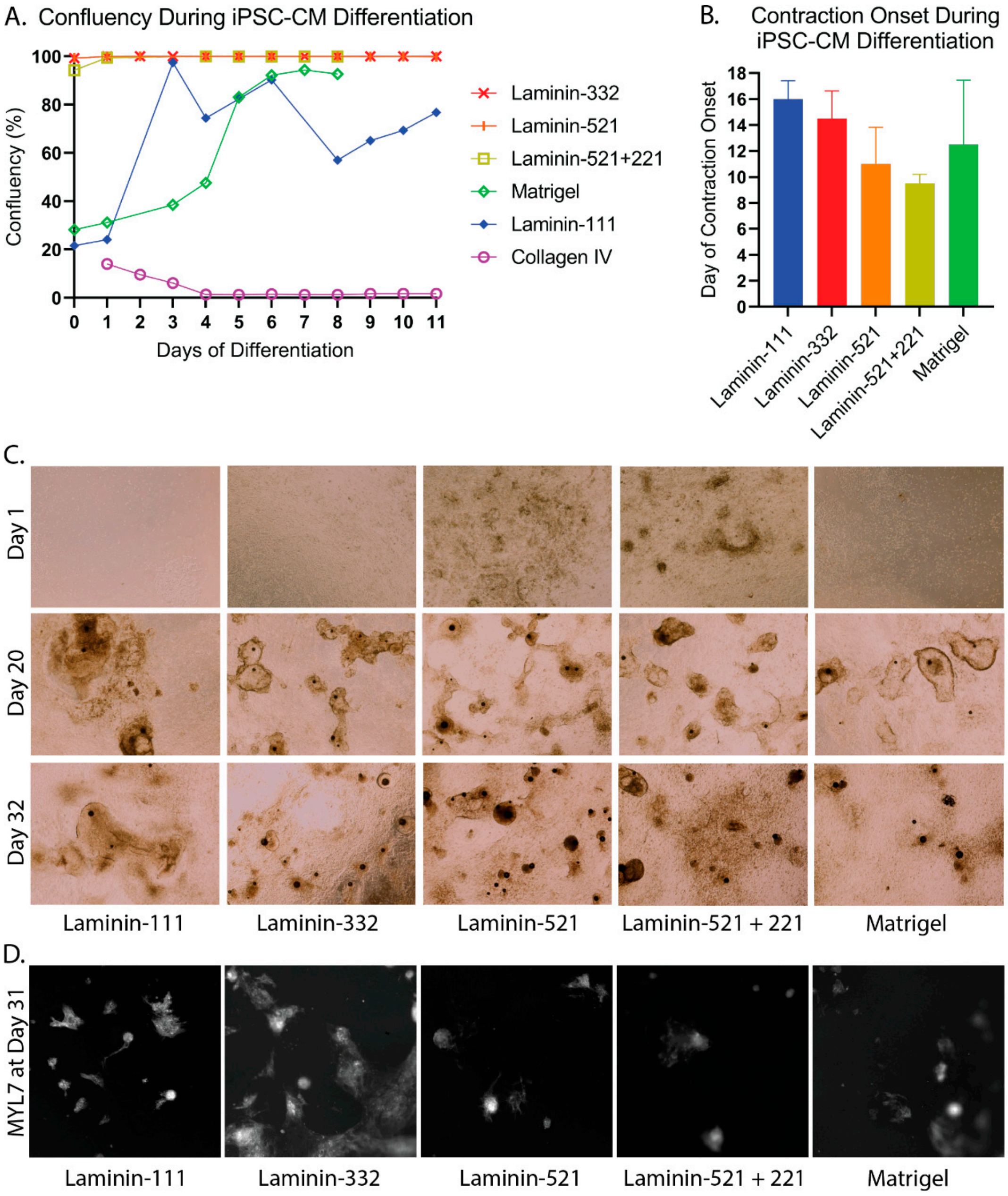

Supplementary Figure S1. Early differentiation of human iPSCs into iPSC-CMs on different ECM proteins. (**A**) Confluency over time during the first 11 days of iPSC-CM differentiation, as imaged and quantified using an Olympus CM20 Provi imaging system, where iPSCs differentiated on human laminins -332, -531, or -531 + -221 (combined) supported the highest levels of confluency, followed by Matrigel or mouse laminin-111, and then mouse collagen IV. Differentiation of iPSCs was also attempted on human laminins -221 and -211 but these substrates did not support iPSC growth (confluency ≈ 0%)(data not shown) (n=1). (**B**) Day of contraction onset during iPSC-CM differentiation, as determined via manual brightfield microscopy inspection, where human laminin-521 alone or in combination with -221 supported an earlier mean onset day compared to Matrigel, and all other ECM proteins tested had a later mean onset day (iPSCs differentiated on mouse collagen IV did not generate contractions during this time) (n=2). (**C**) Brightfield microscopy of iPSCs differentiated on indicated ECM proteins at days 1, 20, and 32 of iPSC-CM differentiation (4X objective magnification). (**D**) Fluorescent microscopy of iPSCs differentiated on indicated ECM proteins at 31 days of iPSC-CM differentiation showing expression of protein MLC-2a (fluorescent tag mEGFP on the *MYL7* gene)(4X objective magnification). Abbreviations: induced pluripotent stem cell-derived cardiomyocyte (iPSC-CM)
